# Evaluating the effects of time-restricted eating on overweight and obese women with polycystic ovary syndrome: A randomized controlled trial study protocol

**DOI:** 10.1371/journal.pone.0316333

**Published:** 2025-01-09

**Authors:** Hui Liu, Fuliang Shangguan, Fen Liu, Yu Guo, Huixi Yu, Hanbing Li, Yinhua Su, Zhongyu Li

**Affiliations:** 1 School of Nursing, Hengyang Medical School, University of South China, Hengyang, Hunan, China; 2 The First Affliated Hospital, University of South China, Hengyang, Hunan, China; University of Montenegro-Faculty of Medicine, MONTENEGRO

## Abstract

**Background:**

Time-restricted eating (TRE) manages weight effectively, but choosing how long and what time window remain debatable. Although an 8:00 a.m. to 16:00 p.m. time frame is reported to show positive results in most weight loss trial, its safety and efficacy in overweight and obese women with polycystic ovary syndrome (PCOS) is uncertain. This randomized controlled trial is conducted to evaluate the safety and efficacy of TRE in specific populations.

**Objective:**

This study aims to assess the 6-month effects of TRE on weight change, metabolic improvement, reproductive recovery, and health-related quality of life in overweight and obese women with polycystic ovary syndrome (PCOS), compared to those who did not receive TRE.

**Methods:**

This randomized controlled trial will enroll 96 overweight and obese women with polycystic ovary syndrome (PCOS), who will be randomly assigned to either a TRE group (with an eating window from 8:00 a.m. to 16:00 p.m.) or a control group (without eating time restrictions), with 49 participants in each group. Evaluators and data analysts will remain blinded to group allocation throughout the study. The primary outcomes, including changes in weight and body mass index (BMI), will be assessed weekly. Secondary outcomes, encompassing alterations in sex hormones, metabolic parameters, body composition, sleep quality, quality of life, anxiety, and depression, will be evaluated monthly. Compliance and safety will be continuously monitored throughout the study. Additionally, a 6-month follow-up will be conducted at the end of the trial to assess the long-term effects of TRE. Statistical analysis will include the Anderson-Darling test for normality, T-test/Wilcoxon test based on distribution, mixed-effects models for assessing time/group effects, Cox model for time-to-event analysis, repeated ANOVA for change analysis, and sensitivity analysis. All tests will be conducted using appropriate software, with a significance level set at P<0.05. Missing data will be imputed.

**Discussion:**

The purpose of this study protocol is to further evaluate the effects of TRE in overweight and obese women with PCOS through a randomized controlled trial (RCT). Findings from this study are expected to provide new dietary intervention strategies for overweight and obese PCOS participants.

**Ethics and dissemination:**

This study has received ethics approval from the Medical Ethics Committee of the University of South China (Number: NHHL027). Participants are included after signing informed consent. Results will be submitted for publication in peer-reviewed journals.

**Trail registration:**

**Trail registration number:** ChiCTR2400086815.

## Introduction

Polycystic ovary syndrome (PCOS) is a common gynecological endocrine disorder with an incidence rate of 5%-18% [1–3]. Globally, approximately 60% of PCOS women are accompanied by overweight or obesity [4]. Previous studies have shown that overweight and obesity are significant risk factors for women with PCOS [5,6]. Overweight and obese women with PCOS amplify conception challenges and lower assisted reproductive technology success rates, while also heightening risks of cardiovascular disease, diabetes, and mental health issues like anxiety and depression [7–10]. Consequently, weight loss is vital for PCOS women to restore fertility, prevent chronic diseases, and enhance their overall well-being [11]. Among weight reduction strategies, dietary intervention stands out as a key management approach [12]. Numerous dietary interventions for PCOS treatment have been suggested, including low-calorie [13], low-carbohydrate [14], low-glycemic index [15], Mediterranean [16], and ketogenic diets [17]. However, these methods face challenges, including adherence, metabolic adaptation, and psychological strain [18]. Time-restricted eating (TRE), an emerging eating pattern, focus on limiting daily eating to specific periods, while completely avoiding calorie-containing intakes during the rest of the day [19]. TRE effectively reduces body weight, lowers blood sugar levels, and improves insulin sensitivity and metabolism by controlling the duration and timing of food intake [20]. Different durations and windows of food intake yield varying effects. 4-hour and 6-hour eating windows reduced patients’ weight and improved cardiometabolic health, but adverse effects such as nausea, headaches, and constipation are reported [21,22]. 10-hour and 12-hour eating windows did not significantly affect weight loss or insulin sensitivity improvement [23–25]. The 8-hour TRE window is most extensive, but results vary based on the specific eating window. Neither too early nor too late time window can achieve the desired effect, or even have a negative impact [26–28]. At present, the time window of 8:00–16:00 works best, which, according to some studies, is consistent with the human body’s circadian rhythm [29–31].

Although TRE has shown good effects such as diabetes, metabolic syndrome, and cardiovascular diseases [32,33], there are limited studies on it in overweight and obese women with PCOS. TRE has shown certain weight loss effects in anovulatory PCOS women [34]. However, due to the small sample size (n = 25), neglecting the impact on ovulation and the non-randomized design, the reliability of the conclusion remains to be verified.

Significant effects of TRE become apparent after 4 weeks of continuous implementation, with the potential for greater weight loss over an extended intervention period of 12 weeks [35,36]. To thoroughly investigate TRE’s impact on this specific demographic, a 12-week randomized controlled trial will be carefully planned. In the trial design, we will combine international health guidelines with the dietary habits of Chinese individuals, ultimately selecting 8:00 a.m. to 16:00 p.m. as the optimal feeding window [37,38]. In addition, to ensure the validity of the trial, calorie intake will be appropriately restricted. Following the conclusion of the trial, a 3-month follow-up will be conducted to further evaluate the long-term effects and safety of TRE. Through such an approach, we aim to comprehensively and precisely evaluate TRE’s efficacy in this population, offering effective, practical, and scientific dietary management strategies to these women, thus helping them achieve positive metabolic health.

## Methods

### Study design and ethics

This prospective randomized controlled study will be used to evaluate the effects of TRE in overweight and obese women with PCOS. After signing the informed consent form, participants will be randomly divided into two groups for a 1-week baseline survey, a 12-week intervention, and a 6-month follow-up. Recruitment for the study began on July 20, 2024, and will end by June 20, 2025. Participants will provide written informed consent. This study was conducted in accordance with the Declaration of Helsinki, and approved by the Medical Ethics Committee of the University of South China (Number: NHHL027), and registered in the Chinese Clinical Trial Registry (ChiCTR 2400086815). If protocol changes are required, the study leader will communicate with ethics Committee members and trial registry staff.

### Setting

The setting for this study encompasses the First Affiliated Hospital of University of South China. Study procedures will be conducted within the outpatient departments of gynecology and nutrition, as well as at the participants’ homes. To avoid interaction between the two groups of participants, the study measurements for the TRE group will be conducted exclusively at their homes. Recruitment will be conducted via flyers, posters, phone calls, social media, and emails.

### Participants

#### Sample size

The study will enroll 96 overweight and obese women with PCOS, and the sample size is based on the estimation formula for the sample size required to compare the two sample means of the measurement data [13]:

$$N=\frac{2*{\left(Z_{1-\frac{\alpha }{2}}+Z_{1-\beta }\right)}^{2}*\sigma^{2}}{\delta^{2}}$$

N represents the sample size for each group. $Z_{1-\frac{\alpha }{2}}$ is the Z-score corresponding to the desired significance level (α). For a significance level of 0.05, $Z_{1-\frac{\alpha }{2}}$ = 1.96. $Z_{1-\beta }$ is the Z-score corresponding to the desired power (1 - β). For a power of 80%, $Z_{1-\beta }$ = 0.84. According to the reviewed literature [39], σ = 3.87, δ = 2.5kg. Considering a 20% to 25% dropout rate, the final sample size will be determined to be 48 participants per group, for a total of 96 participants.

#### Inclusion criteria

(1) age 18–49 years old; (2) a diagnosis of PCOS according to the Rotterdam criteria [40]; (3) BMI ≥ 24 kg/m^2^ (where BMI between 24–27.9kg/m^2^ is overweight, BMI > 28kg/m^2^ is obese); (4) willing to sign a consent form.

#### Exclusion criteria

(1) the participant has used weight loss drugs or hormone therapy in the past six months; (2) body weight fluctuation > 5% in the past three months; (3) on pregnancy, breastfeeding, preparation for pregnancy or perimenopausal period; (4) regular high-intensity exercise or night shifts due to work; (5) serious heart disease or stroke (including myocardial infarction, unstable angina, heart bypass surgery, PTCA, congestive heart failure, and ischemic heart disease) within six months; (6) participants with other diseases (such as primary amenorrhea, thyroid dysfunction, malignant tumors, diabetes, severe gastrointestinal, renal or liver diseases); (7) history of hypotension; (8) participants who have undergone bariatric surgery or have been diagnosed with cancer.

### Termination criteria

(1) incidence of significant security issues; major and unforeseen adverse events directly linked to the intervention; (2) pregnancy; (3) participants requesting to withdraw their informed consent, showing reluctance to comply with the intervention, or due to other unforeseen reasons.

### Randomization and blinding

In this study, randomization will be achieved using opaque closed envelopes and simple random sampling. Based on sample size estimates, two groups will be allocated to a 1:1 ratio. To ensure an unpredictable distribution, 96 random numbers (either 0 or 1, each making up half) will be generated. These numbers will be written on 96 cards and sealed in yellow-brown envelopes for concealed grouping. After unsealing, participants will be assigned to either the “control group” (0) or the “TRE group” (1) based on the random number on the card. Generating the assignment order, recruiting participants, and assigning participants to the intervention will be performed separately by different researchers in this research group.

This study will be an open-label clinical trial design, and participants cannot be blinded because the intervention plan will limit the time of participant eating. To reduce bias error, blinding of outcome assessors and data analysts will be used to compensate. To achieve this, outcome assessors will undergo training to ensure they conduct evaluations in a standardized and objective manner, free from any influence stemming from knowledge of the participant’s group assignment. Additionally, data analysts will work with anonymized datasets, where all identifying information related to group assignment has been removed. Furthermore, stringent protocols will be enforced to prevent any unauthorized communication among outcome assessors, data analysts, and those responsible for assigning participants to groups or delivering interventions.

### Procedures and measures

#### Baseline investigation and development of calorie restriction program

Before the formal trial begins, all potential participants will undergo a 1-week baseline survey. Baseline surveys are designed to confirm participants’ eligibility for the study, assess their interest in the study, and obtain informed consent. At the same time, a series of baseline measures will be taken, such as weight, BMI, body composition, sex hormones, glucose and lipid metabolism indicators, as well as quality of life assessment, sleep quality assessment, anxiety and depression assessment. In addition, a weekly dietary assessment will be conducted using food journals and food photographs to record in detail the participants’ daily eating patterns, food intake and calorie intake. Work intensity and basal metabolic rate will be assessed using the International Physical Activity Questionnaire and a basal metabolic meter, respectively. Based on these assessments, the daily calorie requirements for each participant will be calculated. In the personalized calorie restriction program, each participant will be required not to exceed their individual daily calorie needs and will have the autonomy to decide the types and quantities of food they consume, while ensuring that the overall calorie goal is met.

#### Control group

Participants in the control group will maintain their original eating habits, but will follow a personalized calorie restriction regimen based on baseline survey evaluation results. This means their daily calorie intake will be strictly controlled to ensure they do not exceed the upper limits set out in the programme, promoting healthy weight management. The control group, however, did not undergo a specific TRE intervention, but received regular telephone follow-up visits from researchers to monitor their eating habits and weight changes.

#### TRE group

In addition to following a personalized calorie restriction regimen based on baseline survey assessment results, TRE participants will be instructed to consume their allotted calories within a specific eight-hour time window (8 a.m. to 16 p.m.). During this time, they will have autonomy to decide the specific types and amounts of food they consume, while adhering to their overall calorie targets. Outside of this eight-hour window, participants will be in a fasting state and only allowed to consume sugar-free water and zero-calorie beverages to meet their basic fluid needs. This means that TRE participants will effectively be fasting for 16 hours each day (from 16 p.m. to 8 a.m. the next day).

#### Support and follow-up

To ensure the effective implementation of the intervention and the accuracy of the data, we provided full support and close follow-up for all participants. First, all participants will receive personalized dietary counseling provided by a professional nutritionist. These consultations cover not only the development of meal plans, but also specific guidance on how to carry out these plans in real life. Participants will receive a detailed diet information booklet at the hospital with precise portion size recommendations, sample menus, and practical tips on how to properly schedule meals within a specific time window. In addition, we asked participants to keep a detailed food diary and to take photographs before and after each meal. This practice was designed to help participants better monitor their eating habits, while also providing us with an accurate source of data to assess when they ate and how much food they consumed.

In terms of follow-up, we have developed a strict monitoring plan. During the intervention, two researchers will contact the participants weekly by phone, not only focusing on their diet implementation, but also asking in detail and recording any possible adverse events (such as hunger pangs, dizziness, mood swings, etc.). For any questions or confusion encountered, the researchers will provide timely answers and necessary guidance to ensure participants’ compliance and the smooth conduct of the study. At the same time, participants’ food diaries and food photos will be regularly reviewed, using Chinese food composition lists to accurately assess their nutrient intake [41]. This step is essential to verify the implementation of the diet plan and adjust the diet regimen.

**Fig 1:**
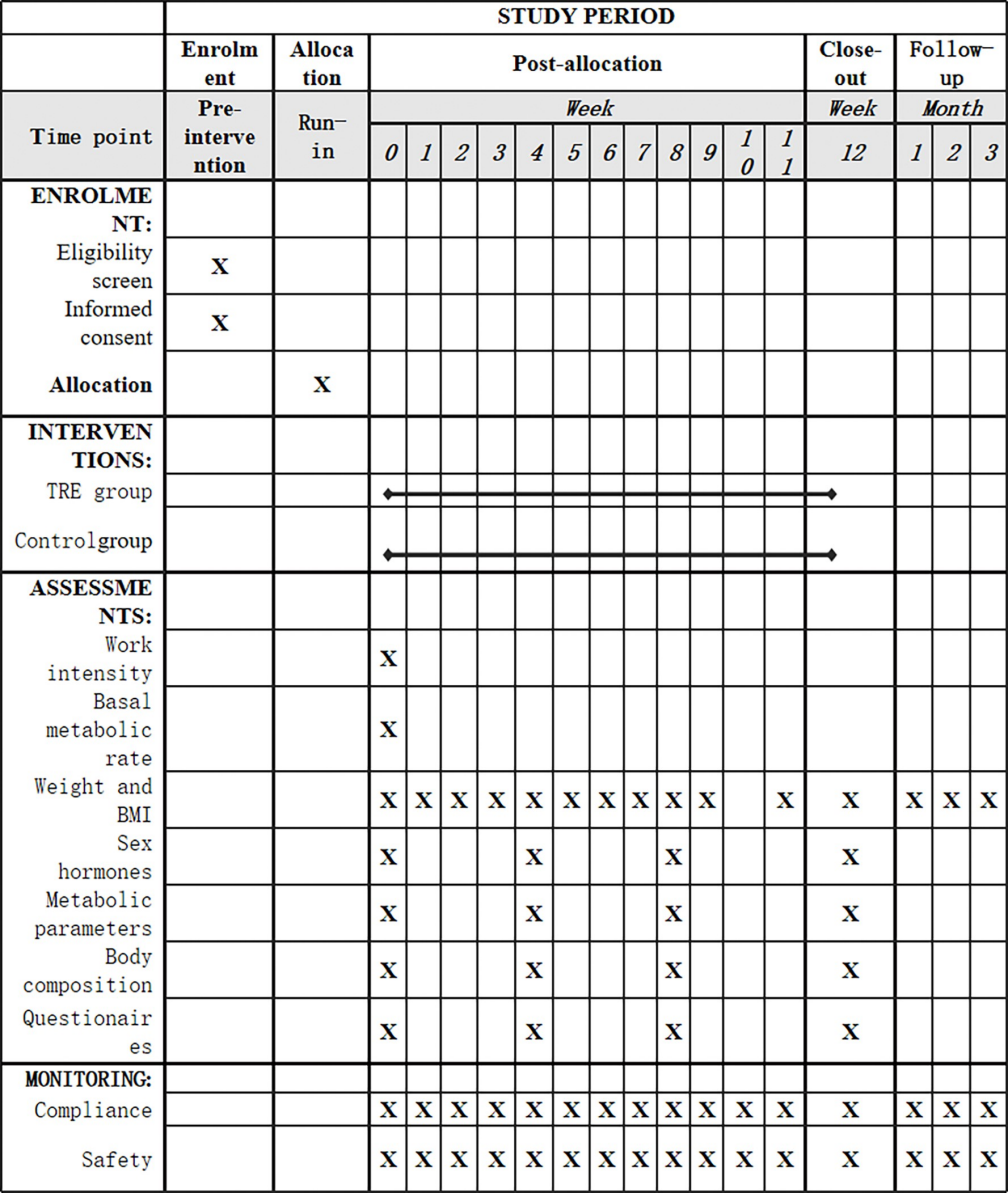
Contents for the schedule of enrolment, intervention and assessments according to SPIRIT requirement.

Our follow-up did not stop after the intervention. Instead, we will continue to contact participants by phone on a monthly basis to check on their weight changes and encourage them to share any new developments regarding their diet, lifestyle habits, or health status. This long-term follow-up mechanism will help us evaluate the long-term effects of time-restricted dietary interventions and provide valuable data to support future studies. Fig 1 shows the schedule for enrolment, intervention and assessment as required by SPIRIT.

### Measures

Study results will be measured at multiple time points and changes from baseline to each time point will be assessed. The results included objective indicators such as weight, sex hormones, metabolism, and subjective indicators such as quality of life [42], sleep quality [43], and psychological status [44]. We will not interfere with the normal treatment of PCOS women throughout the trial period. Table 1 summarized the details of the collection of outcome indicators.

**Table 1:** Outcome measures and measurement methods.

Outcome Measure		Collection Method/Tool			Details	Measurement Time Points
Weight and BMI		Multi-frequency bioelectrical impedance analyzer IOI scanner			Each participant will be asked to take the measurements after fasting (or three hours after eating and drinking) and after emptying their bladder and bowels. Each participant will be asked to take the measurement on an empty stomach (or three hours after eating or drinking water) and after defecating. Participants will take off their shoes and socks during the measurement, and their weight and height will be measured by the multi-frequency bioelectrical impedance analyzer IOI (IOI, Seoul, Korea) scanner to calculate their BMI (weight kg/ height m squared, kg/m^2^).	Intervention: baseline, once a week Follou-up: once a month
Sex hormones		Chemiluminescence immunoassay analysis			Participants will have their sex hormones measured between the 2nd and 4th days of their menstrual cycle; for those with amenorrhea, the date is not restricted. They will fast for 12 hours before the test. The tests will be conducted using chemiluminescence immunoassay analysis.	Baseline, 4^th^ week, 8^th^ week, 12^th^ week
Metabolic parameters		Fasting blood tests			Fasting blood tests will be conducted to analyze glucose metabolism parameters (glucose, insulin, HOMA-IR, and HbA1c levels) and lipid profile (triglycerides, total cholesterol, and LDL/HDL cholesterol).	
Body composition		Multi-frequency bioelectrical impedance analyzer IOI scanner			The measurement requirements are the same as the anthropometry, the body composition will be assessed by the IOI scanner, including body fat (kg), muscle (kg), water (L), inorganic salts (kg), protein (kg).	
**Outcome Measure**	**Collection Method/Tool**			**Details**		**Measurement Time Points**
Scale	Pittsburgh Sleep Quality Index			The Pittsburgh Sleep Quality Index (PSQI) was used to rate participants’ sleep quality. It consists of 19 self-assessment entries and 5 other-assessment entries, in which the 19th self-assessment entry and 5 other-assessment entries are not involved in scoring. 18 entries form 7 components, each component is scored on a 0–3 scale, and the cumulative score of each component is the total PSQI score, which ranges from 0–21, with higher scores indicating poorer sleep quality.		Baseline, 4^th^ week, 8^th^ week, 12^th^ week
	SF-36			Sf-36 consists of eight dimensions: vitality (VT), mental health (MH), physiological function (PF), physiological function (RP), emotional function (RE), physical pain (BP), social function (SF), and general health (GH). The 8 dimensions get their own dimension score according to their own entry score, and the health change score is not included in the total score. The total quality of life score is the sum of eight dimensions, and the higher the total score, the better the quality of life.		
	Hospital Anxiety and Depression Scale			Hospital Anxiety and Depression Scale (HADS) was used to assess the degree of anxiety and depression of the participants, including two subscales, Hospital Anxiety and Depression Scale-Anxiety (HADS-A) and Hospital Anxiety and Depression Scale-Depression (HADS-D), with 7 entries each and a total of 14 entries. Higher scores indicate more severe anxiety or depressive symptoms.		
**Outcome Measure**			**Collection Method/Tool**		**Details**	**Measurement Time Points**
Compliance assessment			*Wechat* app		Compliance will be determined by the number of days participants adhere to the assigned dietary rules. Control group participants will be instructed to limit their caloric intake to the defined daily allotment, while TRE group participants will be consumed within designated mealtimes and meet daily calorie targets. We will employ a combination of methods to monitor compliance, including the use of food diaries and *Wechat* apps that can track meal times, calorie intake, and adherence to dietary rules. To improve participant compliance, each participant will be given a free physical examination and a food scale.	Intervention: baseline, once a week Follou-up: once a month
Safety assessment*			Asked by the investigator or voluntarily reported by the participant		Investigators will collect, record, and report all adverse events that occur during the trial period (beginning with the participant’s informed consent and ending with the participant’s last assessment, whether asked by the investigator or voluntarily reported by the participant). The participant’s symptoms, severity, onset and duration, treatment, and course of action will be recorded in the original data to evaluate their relevance to the trial intervention, and will be recorded in detail, signed and dated by the investigator. If serious adverse events occur during the clinical trial, the investigator will take appropriate treatment measures for the subjects and record them in the original data, and report them to the principal investigator of the trial center and the ethics committee within 24 hours. Researchers will carefully fill in the serious adverse event report form, and sign and date the report.	

*Safety indicators will include: the overall incidence of adverse events and the incidence of each adverse event; the severity and incidence of various types of gastrointestinal reactions, such as nausea and gastroparesis; the severity and incidence of hypoglycaemic events; early withdrawal due to adverse events; changes in vital signs; the severity and incidence of psychoneurological symptoms such as somnolence, anorexia, and other events.

### Data analysis

The Anderson-Darling method will be used to test the normality of continuous variable data. Continuous data will be expressed as M = xx.x, SD = xx.xx (for normal distribution) or median (with interquartile spacing) (for non-normal distribution). Statistical analysis will be performed for either the T-test (normal distribution) or the Wilcoxon rank test (non-normal distribution).

To account for the longitudinal nature of the data and to assess time and group effects, mixed-effects models will be utilized. These models will enable us to capture within-subject variability and more accurately estimate changes over time, particularly for secondary outcomes. Additionally, if time-to-event analysis is relevant (e.g., time to reach certain metabolic or hormonal targets), a Cox proportional hazards model will be included to examine the risk of achieving specific health milestones.

For the analysis of multiple dependent variables simultaneously, controlling for potential confounders such as age or baseline BMI, multivariate analysis of covariance (MANCOVA) will be considered. However, given the complexity and potential limitations (e.g., sample size), the feasibility of this analysis will be carefully assessed. Specifically, we acknowledge that the sample size may be a concern, and we will discuss why certain important factors for weight change (e.g., dietary intake, physical activity levels) were not controlled during sampling. These decisions may have been influenced by practical considerations such as the availability of data or the feasibility of collecting additional information. Despite these limitations, we will explore alternative statistical methods to address these concerns and ensure the robustness of our findings.

Structural equation modeling (SEM) will be explored to provide insights into the interdependencies between various outcomes, such as the relationship between metabolic parameters and psychological outcomes like anxiety or quality of life. A chi-square test for independence will be employed to analyze categorical data, such as compliance levels or adverse events. Repeated measures ANOVA with appropriate post-hoc testing will be utilized to clarify the timing and magnitude of changes, identifying significant differences between time points. Sensitivity analysis will be conducted to assess the robustness of the results by testing different model specifications or excluding outliers.

All statistical analyses will be performed using appropriate software (e.g., GraphPad Prism 8.0.1 or R, depending on the specific requirements of the analysis), and bilateral tests will be used for all results. Statistical significance will be determined at the P < 0.05 level. The intention-to-treat (ITT) population approach will be adopted to handle program non-adherence, ensuring that all randomized participants are included in the analysis according to their original group assignment. Missing data will be addressed using appropriate imputation methods, such as multiple imputation, to minimize bias and maintain the integrity of the statistical analysis.

### Quality control

Pre-intervention: the examination equipment will be thoroughly checked and calibrated to ensure proper functioning and accuracy. All researchers involved in the experiment will receive standardized training and be familiar with the operation methods before implementing the intervention to ensure consistency and accuracy.

During the intervention: operational standards will be strictly enforced and regularly monitored and recorded to ensure consistency and comparability of the experiment. Intervention subjects will receive the intervention in strict accordance with the TRE. Meanwhile, regular data collection and quality audits will be conducted to ensure data accuracy and completeness.

Post-intervention: data will be carefully validated and reviewed. The credibility and reliability of the experimental results will be ensured by reviewing and analyzing the data.

### Confidentiality

Through the signed informed consent, participants are clearly informed of the content, purpose and scope of the collection of their personal information. Ensure that the information collection process complies with relevant laws and regulations and respects the privacy of participants. Throughout the trial, participants’ personal information was anonymized or de-identified to reduce the risk of disclosure. Access to this information is limited to necessary members of the pilot team and third parties (e.g., regulatory authorities, ethics committees) who need to know about participants as required by laws and regulations or as stipulated in the trial agreement.

## Discussion

There is extensive literature suggesting that being overweight and obese can exacerbate symptoms and complications in women with PCOS [45,46]. Weight loss of 5% to 10% is sufficient to improve clinical symptoms such as menstrual disorders and insulin resistance [2]. Given the increased prevalence and severe consequences of overweight and obesity in PCOS, traditional dietary weight loss strategies suffer significant limitations. As TRE is an emerging strategy that can compensate for the shortcomings of traditional dietary interventions and can treat obesity and improve adult metabolism, we will conduct a randomized controlled trial to evaluate the safety and effectiveness of TRE interventions in overweight and obese women with PCOS.

There are strengths of the study protocol. Firstly, we will improve our study protocol and develop a more rigorous randomization, control design and more scientific sample sizes. Our study will adopt a randomized controlled study design, which can effectively prevent selection bias and improve the comparability between groups to ensure the objectivity and accuracy of research results. In the selection of sample size, we will choose a more appropriate calculation formula in order to get more scientific and effective results. Secondly, in terms of outcome measurement, specific indexes of overweight or obese PCOS will be selected to explore the effects of this dietary pattern on metabolic characteristics and even reproductive health. Data will be collected from the same individual at three different time points, which can eliminate inter-individual variance in the results. Thirdly, we combined evidence-based strategy and Chinese eating habits to choose the best eating window, which can improve the compliance of the trial and obtain significant results as well.

However, there are some limitations to our study. First, repeated questionnaires during the trial may induce bias. To avoid memory overlap due to insufficient time intervals, each questionnaire measurement interval will span six weeks, and all participants will complete offline surveys with uniform guidance provided by researchers. Second, this study does not guarantee blindness for study participants, thus introducing potential uncertainty. Nevertheless, we aim to mitigate this through blinded outcome assessors and data analysts.

## Supporting information

S1 Checklist SPIRIT checklist. (DOC)
